# Deletion of the ORF2 gene of the neuropathogenic equine herpesvirus type 1 strain Ab4 reduces virulence while maintaining strong immunogenicity

**DOI:** 10.1186/s12917-018-1563-4

**Published:** 2018-08-22

**Authors:** Christiane L. Schnabel, Christine L. Wimer, Gillian Perkins, Susanna Babasyan, Heather Freer, Christina Watts, Alicia Rollins, Nikolaus Osterrieder, Bettina Wagner

**Affiliations:** 1 Department of Population Medicine and Diagnostic Sciences, College of Veterinary Medicine, Cornell University, Ithaca, NY 14853 USA; 2 Department of Clinical Sciences, College of Veterinary Medicine, Cornell University, Ithaca, NY 14853 USA; 3 Institut für Virologie, Freie Universität Berlin, Philippstrasse 13, 10115 Berlin, Germany

**Keywords:** Horse, EHV-1, ORF2, Virulence factor, Immunoglobulin, Mucosal immune response

## Abstract

**Background:**

Equine herpesvirus type 1 (EHV-1) induces respiratory infection, abortion, and neurologic disease with significant impact. Virulence factors contributing to infection and immune evasion are of particular interest. A potential virulence factor of the neuropathogenic EHV-1 strain Ab4 is ORF2.

This study on 24 Icelandic horses, 2 to 4 years of age, describes the infection with EHV-1 Ab4, or its deletion mutant devoid of ORF2 (Ab4ΔORF2) compared to non-infected controls (each group *n* = 8). The horses’ clinical presentation, virus shedding, viremia, antibody and cellular immune responses were monitored over 260 days after experimental infection.

**Results:**

Infection with Ab4ΔORF2 reduced fever and minimized nasal virus shedding after infection compared to the parent virus strain Ab4, while Ab4ΔORF2 established viremia similar to Ab4. Concurrently with virus shedding, intranasal cytokine and interferon α (IFN-α) production increased in the Ab4 group, while horses infected with Ab4ΔORF2 expressed less IFN-α.

The antibody response to EHV-1 was evaluated by a bead-based multiplex assay and was similar in both infected groups, Ab4 and Ab4ΔORF2. EHV-1 specific immunoglobulin (Ig) G1 was induced 8 days after infection (d8 pi) with a peak on d10–12 pi. EHV-1 specific IgG4/7 increased starting on d10 pi, and remained elevated in serum until the end of the study. The intranasal antibody response to EHV-1 was dominated by the same IgG isotypes and remained elevated in both infected groups until d130 pi. In contrast to the distinct antibody response, no induction of EHV-1 specific T-cells was detectable by flow cytometry after ex vivo re-stimulation of peripheral blood mononuclear cells (PBMC) with EHV-1 in any group. The cellular immune response was characterized by increased secretion of IFN-γ and interleukin10 in response to ex vivo re-stimulation of PBMC with EHV-1. This response was present during the time of viremia (d5–10 pi) and was similar in both infected groups, Ab4 and Ab4ΔORF2.

**Conclusions:**

ORF2 is a virulence factor of EHV-1 Ab4 with impact on pyrexia and virus shedding from the nasal mucosa. In contrast, ORF2 does not influence viremia. The immunogenicity of the Ab4ΔORF2 and parent Ab4 viruses are identical.

**Graphical abstract:**

Graphical abstract – Deletion of ORF2 reduces virulence of EHV-1 Ab4. Graphical summary of the main findings of this study: ORF2 is a virulence factor of EHV-1 Ab4 with impact on pyrexia and virus shedding from the nasal mucosa.
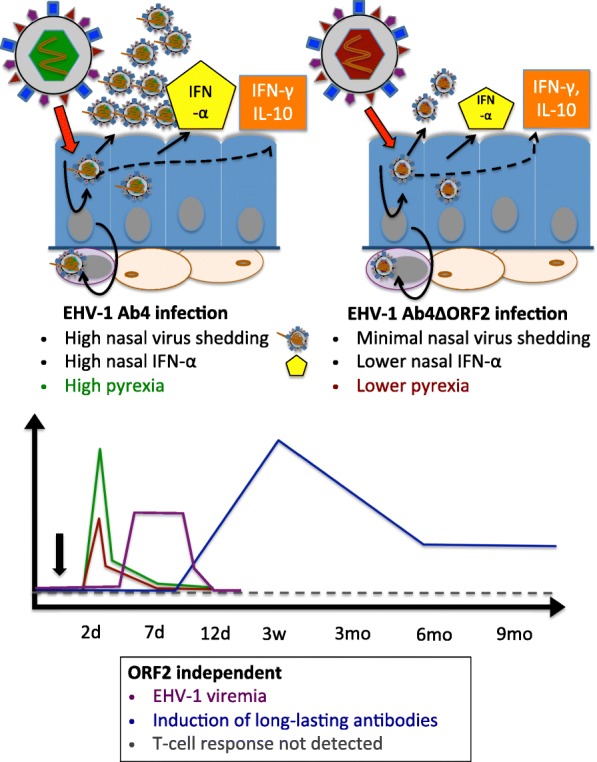

**Electronic supplementary material:**

The online version of this article (10.1186/s12917-018-1563-4) contains supplementary material, which is available to authorized users.

## Background

Equine herpesvirus type 1 (EHV-1), an alphaherpesvirus, frequently induces clinical disease in horses and other equids. Clinical manifestations include upper respiratory tract disease with pyrexia as well as late-term abortions, or neurologic disease (equine herpesvirus myeloencephalopathy, EHM) [1–4]. EHV-1 is transmitted from horse to horse during close contact via respiratory secretions. The virus initially infects the epithelium of the upper respiratory tract. Subsequently, it spreads through the underlying connective tissue to the regional lymphoid tissues, and next establishes cell-associated viremia in leukocytes [5–8]. Viremia is a prerequisite for the infection of endothelial cells by EHV-1 and induction of vasculitis in the pregnant uterus or central nervous system. These events can lead to the severe manifestations of EHV-1, abortions or EHM, respectively [1, 3, 5, 9–11]. Furthermore, EHV-1 establishes latency. Latent EHV-1 is believed to reside in the trigeminal ganglia and in lymphoid tissues of the respiratory tract [12–16].

Latency and immune evasion are two important characteristics of EHV-1 preventing EHV-1 elimination by the immune system of the horse. Latently infected horses serve as a reservoir of the virus. EHV-1 can be reactivated with or without signs of clinical disease in the reservoir horse [17]. Lack of vaccination and incomplete protection after vaccination provide sufficient susceptible hosts within the population for EHV-1 to spread periodically after reactivation from latency or from newly infected horses [4, 17]. The prevalence of EHV-1 is high with detection in 54–88% of horses post mortem [14, 18, 19]. Outbreaks of EHV-1 associated clinical disease occur in variable frequencies but the severity of EHV-1 manifestations seems to be increasing [4, 14, 20]. Consequently, EHV-1 has a significant impact on animal health and the equine economy worldwide and EHM has been classified as an emerging disease in the USA [20, 21]. Management practices and biosecurity measures are applied to contain EHM outbreaks. Many horses are regularly vaccinated in order to prevent infection and disease. However, the use of inactivated or attenuated live virus vaccines has not been sufficient to completely protect from EHV-1 infection and outbreaks [22, 23]. Protective immune responses after vaccination with available vaccines and after natural infection in horses are transient and re-infection is possible between 4 and 8 weeks post vaccination or 6–18 months post infection of adult horses [22, 24–27].

Recent research has focused on the identification of EHV-1 virulence factors such as the mutation of the DNA polymerase gene (ORF30) at nucleotide position 2554, which has been associated with the manifestation of EHM and the establishment of latency after EHV-1 infection. Strains of the genotype G2554 are thus classified as neuropathogenic [2, 28–31]. However, this classification rather points to an increased risk of the G2554 strains causing EHM. During neurological outbreaks between 3 and 45% of the horses developed EHM [32–34] while non-neuropathogenic strains can also cause neurologic disease [2]. The identification and better characterization of virulence factors underlying the pathogenicity, immune modulation, and establishment of latency by EHV-1 can enable targeted approaches to develop a vaccine providing advanced protection from the virus.

In vitro infection of equine peripheral blood mononuclear cells (PBMC) with the non-neuropathogenic EHV-1 strain RacL11 reduced the down-regulation of MHC class I expression [35], and increased interleukin10 (IL-10) secretion [36] and chemokine expression [37] compared to infection with the neuropathogenic strain Ab4. These findings point to differences of EHV-1 strains in modulating the host immune response, which may also be relevant in vivo*.* Potential virulence factors of the Ab4 strain are the ORF1 and ORF2 gene products. ORF1 and ORF2 genes are absent in RacL11, the RacL11-derived modified-live vaccine strain RacH [38], as well as in the apathogenic strain KyA [39, 40]. Hence, the ORF1 and ORF2 genes could encode for immunomodulatory proteins leading to differences in virulence or pathogenicity. The ORF1 gene product pUL56 of Ab4 is responsible for MHC class I down-regulation and seems to be involved in the suppression of cytokine expression and chemokine secretion in vitro [35, 41]. The ORF2 gene is related to ORF1, but is less well characterized. The ORF2 gene product was classified as a membrane protein due to its predicted hydrophobic profile [40]. The deletion of ORF1 and ORF2 genes from EHV-1 Ab4 significantly reduced pyrexia duration and viral shedding compared to the parent Ab4 virus in experimentally infected ponies [42]. This effect could be attributed to either gene or the combination of ORF1 and ORF2.

Here, we performed an experimental infection in horses using a single ORF2 gene deletion mutant of the EHV-1 strain Ab4, Ab4ΔORF2 [35], in comparison to the parent Ab4 virus. The goals of the approach were to identify ORF2 as a potential virulence factor, to characterize the effects of ORF2 on EHV-1 infection and immune responses in horses with known EHV-1 infection and immunity background.

## Methods

### Horses

Twenty-four horses from the Cornell University herd of Icelandic horses with known EHV-1 status were enrolled in this study [43, 44]. All horses were offspring of one stallion, were born at Cornell University, and kept at an isolated facility as a group from birth. They had no contact with other horses in the USA prior to and for the duration of this study. All horses were previously experimentally infected with the EHV-1 strain NY03 after weaning at 7 months of age [44, and unpublished data]. By the time of the present study, the horses were 2–4 years of age and their EHV-1 specific immunity had waned to values typically observed in EHV-1 susceptible horses. EHV-1 specific protective immunity is short-lasting and believed to be maintained for 6–9 months after a single experimental infection [27]. The horses were vaccinated annually against rabies, tetanus, West Nile virus, Eastern and Western Encephalitis virus. They were also dewormed twice a year. Prior to the EHV-1 infection described here, the horses were kept on pasture and were clinically healthy. Grass hay was fed to the horses *ad libitum*. All horses survived and were kept for research purposes at Cornell University after the study.

### Virus preparation

Viruses for experimental infection were EHV-1 Ab4, a neuropathogenic strain isolated from a quadriplegic mare [9, 28, 45] and its ORF2 gene deletion mutant, Ab4ΔORF2, produced by two-step Red-mediated recombination of a recombinant BAC Ab4 as previously described in detail [35]. The viruses were propagated in rabbit kidney cells (RK13) in MEM medium supplemented with 0.292 g/l L-glutamine, 1 mM sodium pyruvate, 100 U/ml Penicillin, 100 μg/ml Streptomycin, 50 μg/ml gentamycin, (all Thermo Fisher Scientific Waltham, MA, USA), and 10% FCS (Atlanta biological, Flowery Branch, GA, USA). Virus titers were determined in RK13 cells as previously described [22, 37].

The identity of each virus preparation was confirmed by conventional PCR on 2 μg of extracted viral DNA, using primers flanking the ORF1 and ORF2 gene region of the EHV-1 strain Ab4: forward primer − 5’-AACAACCCTGGGCTCTTTA -3′ and reverse primer – 5’-GATTCGCACCTCATCTCCAC -3′. The resulting PCR products had a distinct size of 2043 bp for Ab4 and of 1446 bp for Ab4ΔORF2. The deletion of ORF2 in Ab4ΔORF2 was furthermore confirmed by sequencing of the PCR product at the Biotechnology Core Facility of Cornell University. After infection, the titer of each virus preparation was confirmed by titration on a plaque assay as described below.

### Experimental EHV-1 infection

For experimental EHV-1 infection horses were assigned to three groups of eight horses each with four mares and four geldings per group. One group of horses served as non-infected controls (control group) and had a median age of 2 years (range 2–3 years). Horses in a second group were infected with the neuropathogenic EHV-1 strain Ab4 (Ab4 group) and had a median age of 2.5 years (range 2–3 years). The third group was infected with the Ab4ΔORF2 deletion mutant virus (Ab4ΔORF2 group) and had a median age of 2.5 years (range 2–4 years).

One day prior to EHV-1 infection the horses were moved into the isolation barns with individual box stalls and allowed to acclimate. The stalls did not allow the horses to have direct nose-to-nose contact. The non-infected control group was housed in a separate barn and always handled prior to the other groups. The two infected groups were housed in a barn with two separate sections, one for each group, divided by a barrier. Airflow between sections was not possible. Each section had a separate entry with an area for donning personal protective equipment including disposable coveralls, boots, hats, and gloves. Biosecurity precautions were taken and people changed personal protective equipment upon entering and exiting each of the sections to prevent the spread of viruses between the three groups. Within each barn section, the barn had a center hallway with the same airspace and horses were handled in each section as one group. No other specific care was taken to prevent spread of virus from horse-to-horse within each group by animal handlers except for changing gloves after taking nasal swab samples from each horse.

Baseline serum and nasopharyngeal secretion (NS) samples were taken the day before EHV-1 infection (d-1). Baseline physical examination measurements were taken on d-1 and again immediately before EHV-1 infection on d0. Then, Ab4 group horses were infected intranasally with 1 *x* 10^7^ plaque-forming units (PFU) of EHV-1 strain Ab4 in 4 ml of saline with a mucosal atomizer device (Wolfe Tory Medical, Salt Lake City, UT). Horses in the Ab4ΔORF2 group received 1*x*10^7^ PFU of the Ab4ΔORF2 virus in 4 ml saline intranasally. Horses in the control group received 4 ml saline without any virus using the mucosal atomizer device.

### Clinical examination

Rectal temperatures were recorded on d-1, twice daily from d0 until d10 post infection (pi) and then daily on days 11 to 14 pi. Fever was defined as body temperatures exceeding 101.5 °F (38.6 °C). Clinical scores were recorded daily between d-1 and d14 pi by veterinarians according to the grade of depression (0–3), reduced appetite (0–2), nasal discharge (0–6), eye discharge (0–3), mandibular lymph node swelling (0–3) and ataxia (0–5). To determine ataxia, neurologic examinations were performed as previously described [46]. The gait was evaluated by walking the horses individually in the aisle, including turns to both sides. A summarized clinical score for each horse and day was calculated as the sum of the scores (possible range 0–22). Due to the housing arrangement examiners were aware of the identity of the control group, but blinded to the identification of the Ab4 or Ab4ΔORF2 groups. The control group was always worked with first.

### Sample collection and processing

The horses were assigned randomized ID numbers, which did not reveal the group affiliation to the laboratory personnel. Samples were processed based on ID numbers and the group affiliation remained blinded until data collection and assay evaluation was completed.

NS were acquired on d-1, and d1-d9 pi using two polyester tipped applicators (Puritan Medical Products Company, Gullford, Maine, USA) swabbed on the nasal mucosa of one nostril for 5 s. Swabs were immediately put into 1 ml of sterile phosphate buffered saline (PBS) in a polystyrene tube. The swabs (in PBS) were transported to the laboratory, maintained at 4 °C and processed within 2 hours after sampling. For virus isolation, NS samples were not further processed. For the analysis of intranasal cytokines and antibodies, NS samples were spun at 1000×g at 4 °C for 5 min leaving the swabs in the collection tube. The diluted NS surrounding the swabs was collected into a 1.5 ml centrifuge tube and the cleared fluid supernatant was stored at − 20 °C until analysis.

Blood samples were collected on d-8, d-1, d1 to d14, d24, d60, d100, d130, d170, d220, and d260 pi by jugular venipuncture with a vacutainer system (20G Monoject Covidien, Medtronic Minneapolis, MN, USA) into glass tubes without anticoagulant or with sodium heparin (both BD, Franklin Lakes, NJ, USA). Serum and heparinized blood samples were processed as described previously [43, 44].

Within 3 h after blood collection PBMC were isolated by density gradient centrifugation (Ficoll-Paque Plus, GE Healthcare, Piscataway, NJ, USA) and re-suspended in cell culture media (DMEM supplemented with 1% (*v*/v) non-essential amino acids, 2 mM L-glutamine, 50 μM 2-mercaptoethanol, 50 μg/ml Gentamicin, 100 U/ml penicillin, 100 μg/ml streptomycin, all from all Thermo Fisher Scientific Waltham, MA, USA and 10% FCS, Atlanta biological, Flowery Branch, GA, USA) at 6 × 10^6^ cells/ml for the following experiments. For PCR detection of viremia, 1 × 10^7^ PBMC were washed twice in PBS, dry cell pellets were snap frozen in liquid nitrogen, and stored at − 80 °C until PCR was performed.

Serum samples were stored in aliquots at − 20 °C until analysis.

### Virus isolation

To determine nasal virus shedding virus isolation was performed as plaque assays as previously described [22]. Briefly, vortexed NS were titrated on confluent RK13 cells. Virus penetration into the cells was allowed during an incubation for 2-4 h at 37 **°**C before supernatants were aspirated and an overlay with 0.5% Methylcellulose (Sigma Aldrich, St. Louis, MO, USA) in cell culture medium (Gibco MEM medium, supplemented with 0.292 g/l L-glutamine, 1 mM sodium pyruvate, 50 μg/ml Gentamicin, 300 U/ml Penicillin, 300 μg/ml Streptomycin, 0.75 μg/ml Amphotericin B, all from Thermo Fisher Scientific and 10% inactivated FCS, Atlanta biological) was performed. After 5 days of incubation, plaques were counted after fixation and staining in crystal violet staining solution (PBS containing 4% *v*/v Paraformaldehyde, 1% *v*/*w* Methanol, 0.05% *w*/*v* Crystal Violet, all Sigma Aldrich). Viral titers were expressed as PFU per ml NS.

### EHV-1 PCR

Cell associated viremia was evaluated by real-time PCR targeting the gB gene of EHV-1 performed exactly as previously described [44, 47]. A total of 1 × 10^7^ PBMC was used for DNA extraction per horse and time point. The PCR was performed at the Animal Health Diagnostic Center at Cornell University.

### EHV-1 multiplex assay

EHV-1 specific antibodies in serum and NS samples were determined using a fluorescent bead-based EHV-1 multiplex assay. The assay was previously described in detail [43] and has been slightly modified for this approach: Instead of coupling viral glycoproteins directly to the beads a monoclonal anti-equine IL-4 antibody (clone 25, [48]) was coupled to all three beads numbered 33, 35 and 36 (Luminex Corp., Austin, TX, USA). Bead 33 was then incubated with IL-4/EHV-1 glycoprotein B (gB), bead 35 with IL-4/EHV-1 gC, and bead 36 with IL-4/EHV-1 gD. All three IL-4/EHV-1 glycoproteins were expressed as described previously [43]. The EHV-1 gB, gC and gD coated beads were then multiplexed, and incubated with diluted serum (1:400) or undiluted NS samples, followed by the respective detection antibodies as described [43]. Total EHV-1 specific immunoglobulin (Ig) was detected by biotinylated goat anti-horse IgG (H + L) antibody (Jackson Immunoresearch Laboratories, WestGrove, PA, USA). Ig isotype detection was performed by biotinylated monoclonal antibodies specific for IgG1 (CVS45) and IgG4/7 (CVS39) [both [49]], IgG1/3 (clone 159–4), IgG3/5 (clone 586), IgG6 (clone 267) [all three [50]], and IgA (BVS2) [51]. Data were reported as median fluorescent intensities (MFI).

### Ex vivo re-stimulation of PBMC with EHV-1

EHV-1 re-stimulation of PBMC was performed as previously described in detail [36, 44, 52]. In brief, PBMC were re-stimulated ex vivo with EHV-1 strain Ab4 at a multiplicity of infection of 1 for 48 h. As controls culture in medium alone or stimulation with Phorbol12-myristate13-acetate (PMA; 25 ng/ml) and ionomycin (1 μM; both Sigma Aldrich) were included. Supernatants were collected for the analysis of secreted cytokines. In assays for intracellular staining of cytokines and flow cytometric analysis, protein secretion was blocked by Brefeldin A (10 μg/ml; Sigma Aldrich) added for the last 24 h of incubation, before the cells were fixed.

### Multiplex assay detection of cytokine secretion

Equine cytokines were quantified in PBMC supernatants, serum and NS samples using a fluorescent bead-based multiplex cytokine assay as previously described [53]. Interferon (IFN)-α, IL-4, and IL-10 were reported in pg/ml and IFN-γ, and IL-17 were reported as U/ml. Furthermore, soluble cluster of differentiation 14 (sCD14), an inflammatory marker, was determined in serum and NS samples by a separate bead-based assay as previously described in detail [54] and was reported in ng/ml. All samples were measured undiluted except for the sCD14 analysis in serum. The latter samples were diluted at 1:200.

### EHV-1-specific T-cell detection by flow cytometry

Flow cytometric analysis of EHV-1 specific cells was performed as previously described [43, 44, 52]. In brief, after EHV-1 re-stimulation PBMC were fixed, permeabilized, and triple stained for CD4, and CD8 on the cell surface and intracellular IFN-γ production or intracellular IL-10, IL-17A, and IL-4, respectively. For each sample 100,000 events were analyzed in a FACS Canto II flow cytometer (BD Biosciences, San Diego, CA). The evaluation of the data was performed using FlowJo version 10.2 (FlowJO LLC, Ashland, OR, USA). A small lymphocyte gate was set by morphology and the proportions of IFN-γ positive lymphocytes after re-stimulation, compared to medium controls were assessed as previously described [52]. The average intracellular cytokine detection in PMA/ionomycin stimulated PBMC (positive controls) was 14.7% IFN-γ positive, 2.2% IL-4 positive, 2.2% IL-10 positive, and 0.1% IL-17 positive lymphocytes.

### Statistical analysis

The data sets of this study were not normally distributed according to D’Agostino and Pearson normality tests. All data sets were analyzed by repeated measures ANOVA with Tukey’s Post-hoc test to identify group differences for each time point analyzed. *P*-values < 0.05 were considered significant. Correlation of peak virus shedding and peak nasal cytokine concentrations were analyzed by two-tailed Spearman rank correlation. The statistical analysis was performed using GraphPad Prism software version 6 or higher (GraphPad Software Inc., La Jolla, CA, USA).

## Results

### Clinical signs after EHV-1 infection

**Fig. 1:**
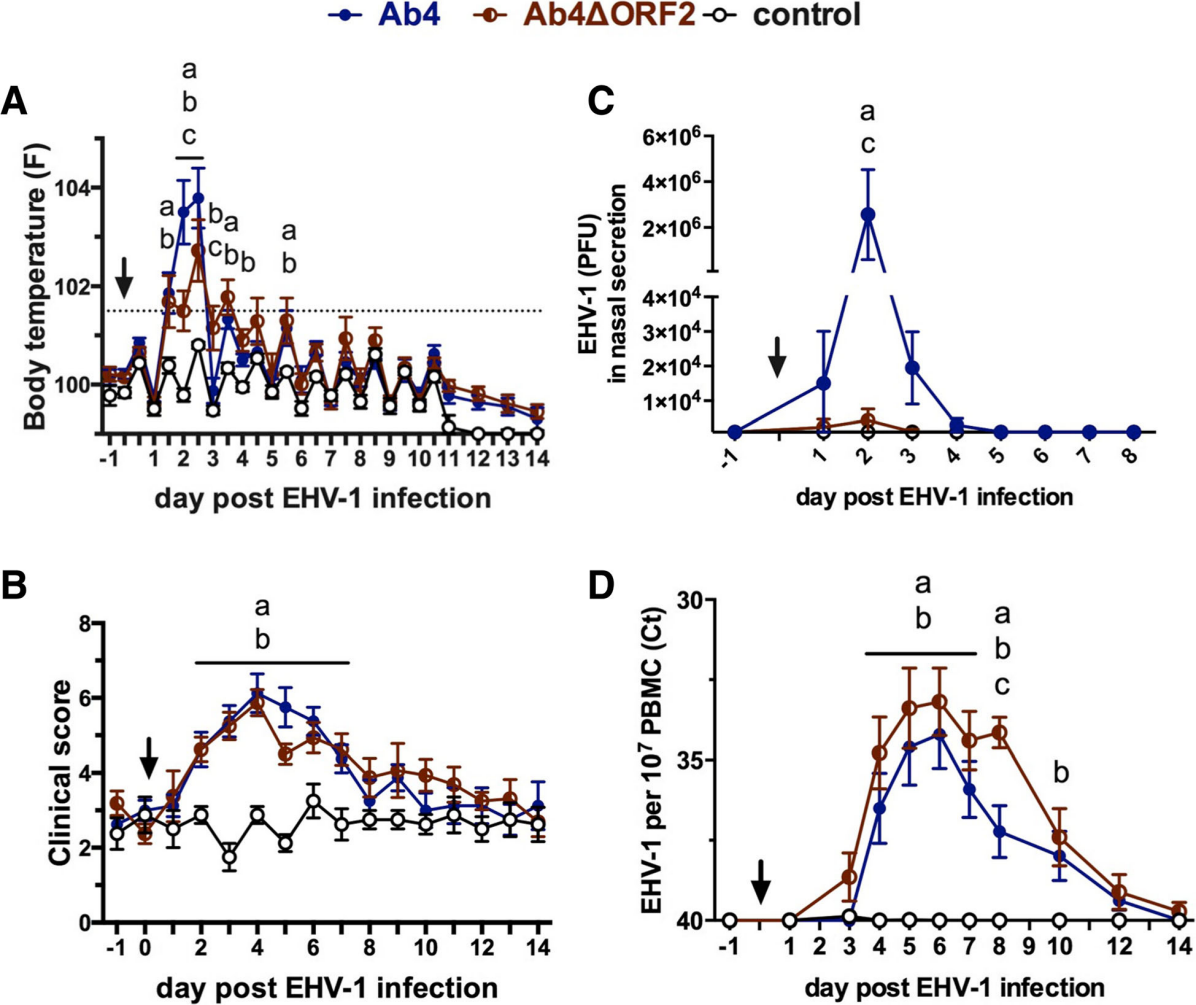
Clinical findings and virus detection after experimental infection with the EHV-1 strain Ab4 or its deletion mutant Ab4ΔORF2. Three groups of horses (*n* = 8/group) were either not infected (control), infected with the neuropathogenic EHV-1 strain Ab4 (Ab4) or infected with its ORF2 deletion mutant (Ab4ΔORF2) on day 0 (arrow). Clinical signs were monitored and virus detection was performed before (day − 1) and after infection (day 1 to 14). Body temperature (**a**); clinical score calculated as the sum of numerical scores for nasal discharge, ocular discharge, lymph node enlargement, ataxia, depression and reduced appetite ranging from 0 to 22 (**b**); viral shedding expressed as plaque forming units (PFU/ml) detected by virus isolation in nasopharyngeal secretions (**c**); viremia determined in 1 × 10^7^ PBMC by real time PCR and shown as Ct values for the gB gene (**d**) were analyzed; Mean and standard errors are plotted for each group. Dotted horizontal lines represent a cutoff of fever at 101.5 °F (**a**) and EHV-1 detection at a Ct 38 (D). Significant differences between groups are marked: **a** = Ab4 vs. control, **b** = Ab4ΔORF2 vs. control, **c** = Ab4 vs. Ab4ΔORF2

Horses in both infected groups, Ab4 and Ab4ΔORF2, displayed clinical signs of mild upper respiratory tract disease as well as fever, while horses in the control group did not (Fig. 1a and b). Horses in the Ab4 group developed an initial fever peak from d1.5 to d2.5 pi, which was significantly higher than the control group (*p* < 0.0001). The initial fever peak was less pronounced in the Ab4ΔORF2 group. Although Ab4ΔORF2 horses had increased body temperatures compared to the control group on d1.5 (*p* < 0.001), d2 and d2.5 pi (both *p* < 0.0001), the fever in the Ab4ΔORF2 group was lower than in the Ab4 group on d2 (*p* < 0.001) and d2.5 pi (*p* < 0.01). Afterwards, body temperatures in both infected groups were significantly increased compared to the control group at several time points until d5 pi (Fig. 1a).

Horses in the control group had low clinical scores between 0 and 5 (median 3) resulting from mild nasal or ocular discharge while stabled. In both infected groups, nasal discharge and enlarged submandibular lymph nodes were the most prevalent clinical signs of disease. Clinical scores in the Ab4 and Ab4ΔORF2 groups were similar over the course of the study and higher than in the controls on days 2 to 7 pi (Fig. 1b). Mild signs of ataxia (score 1) were seen in one horse each in the infected groups. These were noted in the Ab4 group on days 9 and 10 pi and in the Ab4ΔORF2 group on days 5, 6 and 9 pi, respectively. These symptoms resolved completely in both horses by d10 pi.

### Viral shedding and viremia after EHV-1 infection

Virus was isolated from the NS samples of horses in the infected groups between d1to d6 pi and peaked on d2 pi (Fig. 1c). Viral shedding on d2 pi was reduced in Ab4ΔORF2 infected horses compared to those in the Ab4 group (*p* < 0.0001). Although viral shedding was detected in horses of the Ab4ΔORF2 group, their NS virus amounts were low and not different from the control group throughout the study. No virus was detected in NS of the control group.

Cell-associated viremia was detected by PCR. Viral DNA was detectable in PBMC of both infected groups and significantly elevated compared to the control group between days 4 to 8 pi (*p* < 0.01, Fig. 1d). On d8 pi, higher amounts of viral DNA were detected in PBMC of the Ab4ΔORF2 group than in those of the Ab4 group (*p* < 0.01) and on d10 pi viral DNA was still elevated in this group compared to the control group (*p* < 0.01). Viremia was not detected in the control group.

### Intranasal cytokine production in response to EHV-1 infection

**Fig. 2:**
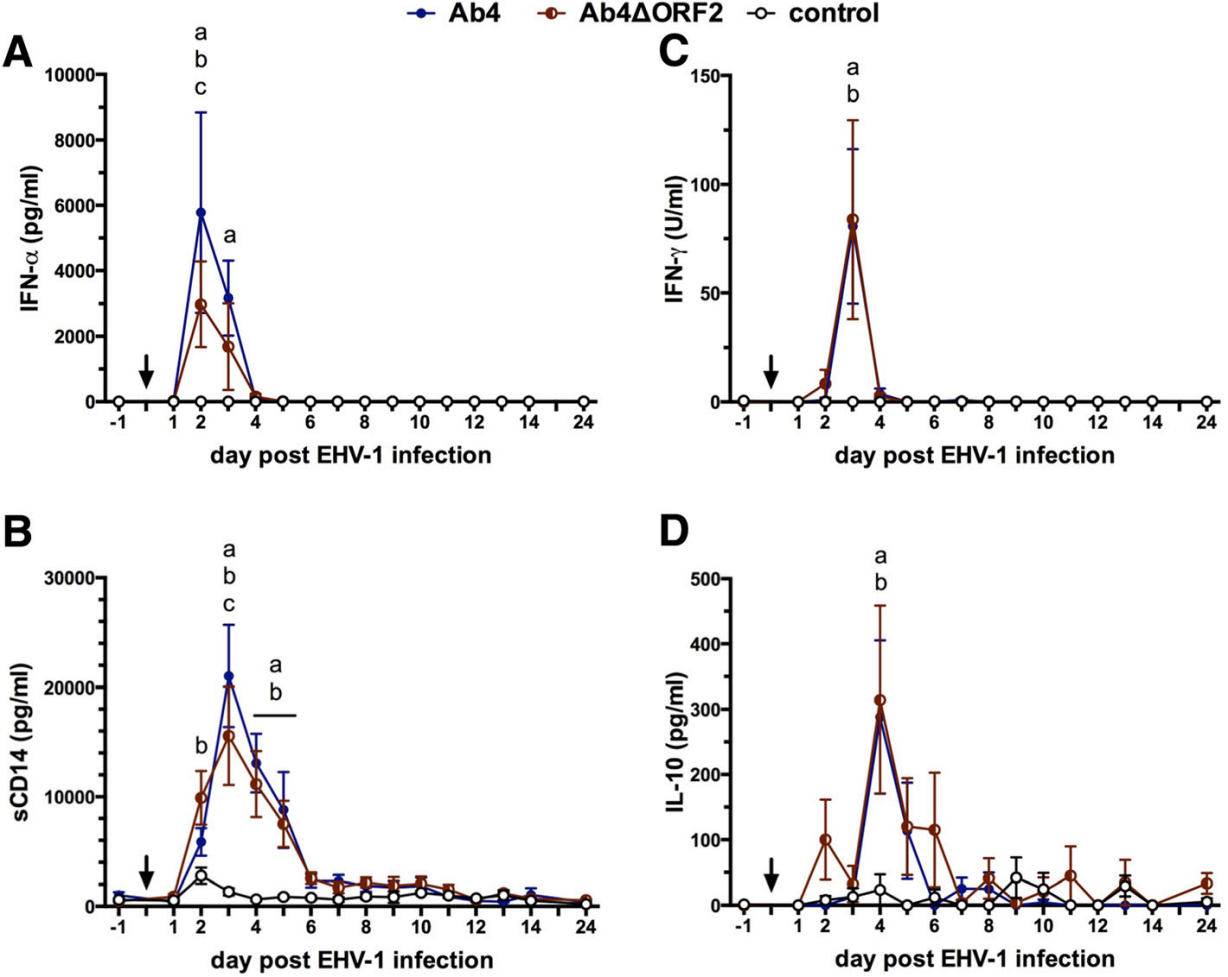
Intranasal cytokine secretion after infection with EHV-1 strain Ab4 or its deletion mutant Ab4ΔORF2. Three groups of horses (*n* = 8/group) were either not infected (control), infected with the neuropathogenic EHV-1 strain Ab4 (Ab4) or infected with its ORF2 deletion mutant (Ab4ΔORF2) on day 0 (arrow). Before (day − 1) and after (day 1–24) experimental infection IFN-α (**a**), sCD14 (**b**), IFN-γ (**c**), and IL-10 (**d**) were quantified in nasopharyngeal secretions by fluorescent bead-based assays. Mean and standard errors are plotted for each group. Significant differences between groups are marked: **a** = Ab4 vs. control, **b** = Ab4ΔORF2 vs. control, **c** = Ab4 vs. Ab4ΔORF2

Within the first days after intranasal infection with Ab4 or Ab4ΔORF2 cytokines were detected in the NS. As the first cytokine, IFN-α was detected and peaked in both infected groups at d2 pi, (Fig. 2a) which corresponds with the time of maximum nasal virus shedding (Fig. 1c). The intranasal IFN-α concentration of the Ab4 group exceeded that of the Ab4ΔORF2 group on d2 pi (*p* < 0.001) and it remained significantly higher than in the control group on d3 pi (*p* < 0.001). IFN-α concentrations declined to undetectable values by d4 pi. Intranasal IFN-α was not detected in the control group.

The inflammatory protein sCD14 increased in NS of both infected groups starting on d2 pi to peak on d3 pi, and remained elevated compared to the control group until d5 pi (Fig. 2b). In Ab4ΔORF2 infected horses sCD14 was increased on d2 pi in comparison to the control and Ab4 groups (both *p* < 0.001), while the peak sCD14 concentration on d3 pi was higher in Ab4 infected than in Ab4ΔORF2 infected horses (*p* < 0.05).

Nasal IFN-γ increased as a sharp peak on d3 pi in the Ab4 and Ab4ΔORF2 groups while it remained undetectable in the control group. Differences in IFN-γ values between the Ab4 and Ab4ΔORF2 infected groups were not observed (Fig. 2c). At d4 pi IL-10 concentrations peaked in NS of both infected groups compared to the control group (*p* > 0.0001) and declined gradually to baseline values by d6 pi in the Ab4 or d7 pi in the Ab4ΔORF2 group. Differences in IL-10 values between the Ab4 and Ab4ΔORF2 infected groups were not observed (Fig. 2d). Intranasal IL-4 and IL-17 were overall low or not detectable and similar between all three groups (data not shown).

The maximum values of virus shedding on d2 pi positively correlated with the peak values of nasal IFN- α and sCD14 (Table 1).

**Table 1:** Spearman rank correlations of peak nasal cytokines and sCD14 with maximum virus shedding on day 2 pi

day pi	IFN-α	sCD14	IFN-γ	IL-10
	d2 pi	d3 pi	d3 pi	d4 pi
r	0.8066	0.7866	0.6442	0.4860
95% CI^a^	0.5894 to 0.9150	0.5526 to 0.9056	0.3140 to 0.8354	0.09026 to 0.7492
*P* value	< 0.0001	< 0.0001	0.0007	0.0160

^a^*CI* confidence interval

### Antibody response to infection

**Fig. 3:**
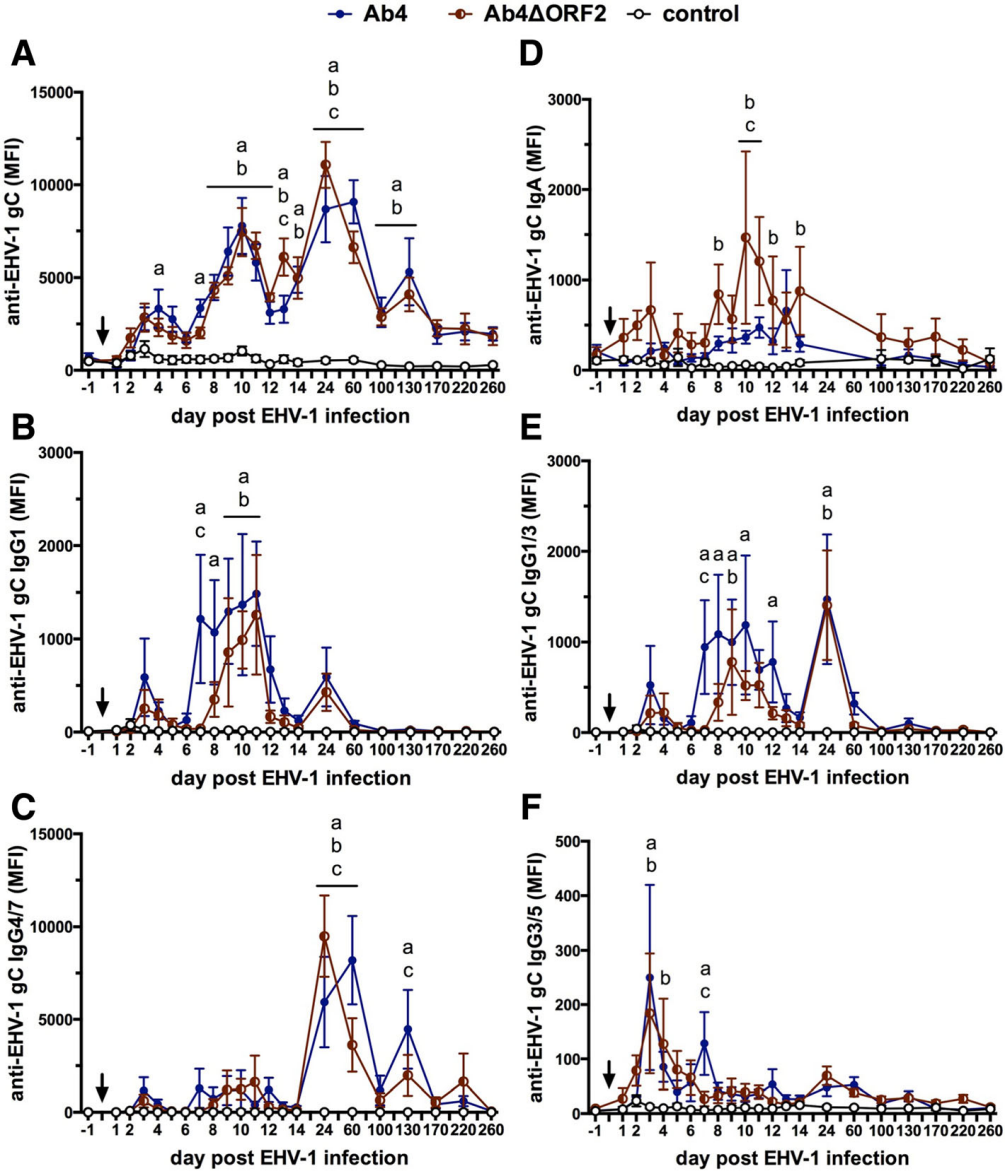
Anti-EHV-1 gC antibodies in nasal secretions after infection with EHV-1 strain Ab4 or its deletion mutant Ab4ΔORF2. Three groups of horses (*n* = 8/group) were either not infected (control), infected with the neuropathogenic EHV-1 strain Ab4 (Ab4) or infected with its ORF2 deletion mutant (Ab4ΔORF2) on day 0 (arrow). Before (day − 1) and after (day 1–260) experimental infection EHV-1 gC specific total Ig (**a**), IgG1 (**b**), IgG4/7 (**c**), IgA (**d**), IgG1/3 (**e**), and IgG3/5 (**f**) were quantified in nasopharyngeal secretions by fluorescent bead-based multiplex assays and expressed as median fluorescent intensities (MFI). Note that Y-axes scales differ depending on isotype. Mean and standard errors are plotted for each group. Significant differences between groups are marked: **a** = Ab4 vs. control, **b** = Ab4ΔORF2 vs. control, **c** = Ab4 vs. Ab4ΔORF2

Antibodies specific for EHV-1 gB, gC, and gD were determined before and following infection in NS and serum. The antibody responses to the three EHV-1 antigens were slightly different in magnitude (gB = gD < < gC) but followed a similar overall pattern. Thus, representative results of EHV-1 gC-specific antibodies are presented here. Total EHV-1 gC-specific Ig in NS and serum were low before infection (d-8, d-1) in all horses and EHV-1 specific antibody induction was not detected in the control group throughout the study (Fig. 3).

#### Intranasal antibody response to infection

Infection with either virus, Ab4 or Ab4ΔORF2, induced EHV-1 gC-specific intranasal antibodies following a wave-like pattern, characterized by several peaks on days 3–4, 10, 24–60, and 130 pi (Fig. 3a). The highest magnitude of the anti-gC antibody response was observed at d24 pi (Ab4ΔORF2 group) or d60 pi (Ab4 group). The increase of total anti-gC antibodies was significantly higher in both infected groups compared to the control group from days 8 to 130 pi, and in the Ab4 group also on days 4 and 7 pi. After d170 pi, nasal EHV-1 specific antibodies in both infected groups returned to low values until the end of the study period on d260 pi, which were not significantly different from the control group. Intranasal gC-specific antibodies were higher in the Ab4ΔORF2 group than in the Ab4 group on d13 pi (*p* < 0.01) and d24 pi (*p* < 0.05), while the Ab4 group exceeded the Ab4ΔORF2 group on d60 pi (*p* < 0.05) (Fig. 3a).

The initial intranasal antibody increase on d3-4pi was composed of low amounts of different gC-specific IgG isotypes, and in the Ab4ΔORF2 group also some IgA (Fig. 3b-f). Afterwards, the intranasal anti-gC total antibody peaks (Fig. 3a) were each dominated by different isotypes. The second anti-gC peak on day 10 pi corresponded to an increase of primarily IgG1 in both infected groups (Fig. 3b). IgG1 was induced slightly earlier (d7, *p* < 0.001) in the Ab4 than in the Ab4ΔORF2 group (d8). Similarly, the third anti-gC antibody peak on days 24 (Ab4ΔORF2) and 60 pi (Ab4), respectively, and the fourth peak on d130 pi were mainly composed of IgG4/7 (Fig. 3c). Anti-gC IgG4/7 antibodies almost mimicked the total anti-gC response shown in Fig. 3a from d24 pi on. This included the higher IgG4/7 in the AbΔORF2 compared to the Ab4 group on d24 pi (*p* < 0.01), the lower intranasal IgG4/7 in the AbΔORF2 group on d60 pi (*p* < 0.0001), and on d130 pi (*p* < 0.05) compared to the Ab4 group.

Notably, intranasal gC-specific IgA after infecting horses with the Ab4 virus was not different from the control horses at any time after infection. In contrast, gC-specific IgA was increased in the Ab4ΔORF2 group compared to the control group on days 8, 10 to 12 and 14 pi. It was also higher than in the Ab4 group on d10 and d11 pi (Fig. 3d). Overall the intranasal EHV-1 specific IgA response in the Ab4ΔORF2 group appeared to be of low magnitude and short duration.

EHV-1 gC-specific IgG1/3 induction mimicked IgG1 antibodies until d14 pi (Fig. 3e). IgG3/5 antibodies were overall low including the early peak on d3pi (Fig. 3f). This supports that the IgG1/3 antibody response until d14 pi was mainly composed of IgG1. On d24 pi, an increase in IgG1/3 antibodies was observed in both infected groups (*p* < 0.0001). This increase was higher than the corresponding IgG1 response in Fig. 3b and likely results from a mixture of anti-gC IgG1 and IgG3 on d24 pi.

EHV-1 specific IgG6 in nasal secretion was low for all groups throughout the study and no group differences were observed (data not shown).

#### Serum antibody response to EHV-1 infection

**Fig. 4:**
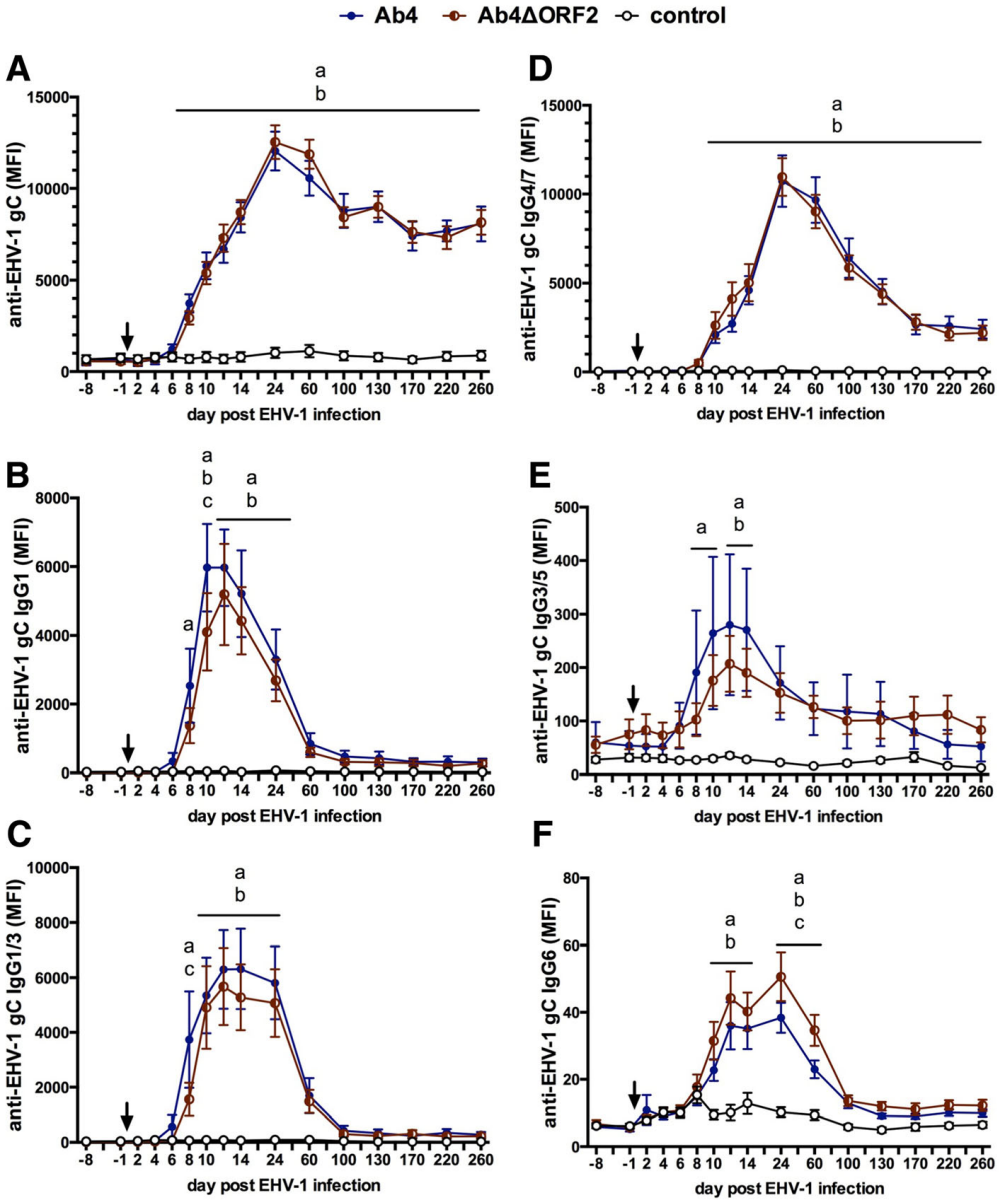
Anti-EHV-1 gC antibodies in serum after EHV-1 infection. Three groups of horses (*n* = 8/group) were either not infected (control), infected with the neuropathogenic EHV-1 strain Ab4 (Ab4) or infected with its ORF2 deletion mutant (Ab4ΔORF2) on day 0 (arrow). Before (day − 1) and after (day 1–260). EHV-1 gC specific total Ig (**a**), IgG1 (**b**), IgG1/3 (**c**), IgG4/7 (**d**), IgG3/5 (**e**) and IgG6 (**f**) were quantified in serum by fluorescent bead-based multiplex assays and expressed as median fluorescent intensities (MFI). Note that Y-axes scales differ depending on isotype. Mean and standard errors are plotted for each group. Significant differences between groups are marked: **a** = Ab4 vs. control, **b** = Ab4ΔORF2 vs. control, **c** = Ab4 vs. Ab4ΔORF2

Infection with either Ab4 or Ab4ΔORF2 resulted in the induction of EHV-1 gC-specific Ig in serum which was first increased on d8 pi compared to the control group (*p* < 0.01). Anti-gC antibodies peaked on d24 pi, slowly declined until d170 pi, and then remained constant in both infected groups for the remainder of the study (Fig. 4a) with *p*-values< 0.0001 between days 10 to 260 pi for both infected groups in comparison to the control group. Total gC-specific Ig was similar between the Ab4 or Ab4ΔORF2 groups for the duration of the study and until d260 pi.

In both infected groups, Ab4 and Ab4ΔORF2, IgG1 (Fig. 4b) and IgG1/3 isotypes (Fig. 4c) were detectable early after infection. They peaked on d10 or d12 pi and declined by d60 pi to baseline values. In the Ab4 group, IgG1 and IgG1/3 induction was slightly earlier than in the Ab4ΔORF2 group (d8 vs. d10) and reached higher IgG1 values on d10 pi (*p* < 0.05).

Anti-gC IgG4/7 antibodies were first detectable on d10 pi in both infected groups and peaked on d24 pi. IgG4/7 primarily contributed to the long-term EHV-1 specific antibody response with both infected groups exceeding the antibody values of the control group from d10 until d260 pi (*p*-values between *p* < 0.0001 and *p* < 0.05) (Fig. 2d). Differences in the IgG4/7 response of the Ab4 and Ab4ΔORF2 groups were not observed.

EHV-1 specific IgG3/5 was weakly induced and elevated in comparison to the control group in the Ab4 group on days 8 to 14 pi (*p* < 0.05 to *p* < 0.001), and in the Ab4ΔORF2 group on d12 and d14 pi (both *p* < 0.05) (Fig. 4e). IgG6 was present in low quantities, which were increased compared to the control group in the Ab4 and Ab4ΔORF2 group on days 10–60 pi (*p* < 0.05). On days 24 and 60 pi the IgG6 antibodies in the Ab4ΔORF2 group exceeded those in the Ab4 group (both *p* < 0.05) (Fig. 4f). EHV-1-specific IgA was not detected in serum at any time point (data not shown).

### Cellular immune response to EHV-1

**Fig. 5:**
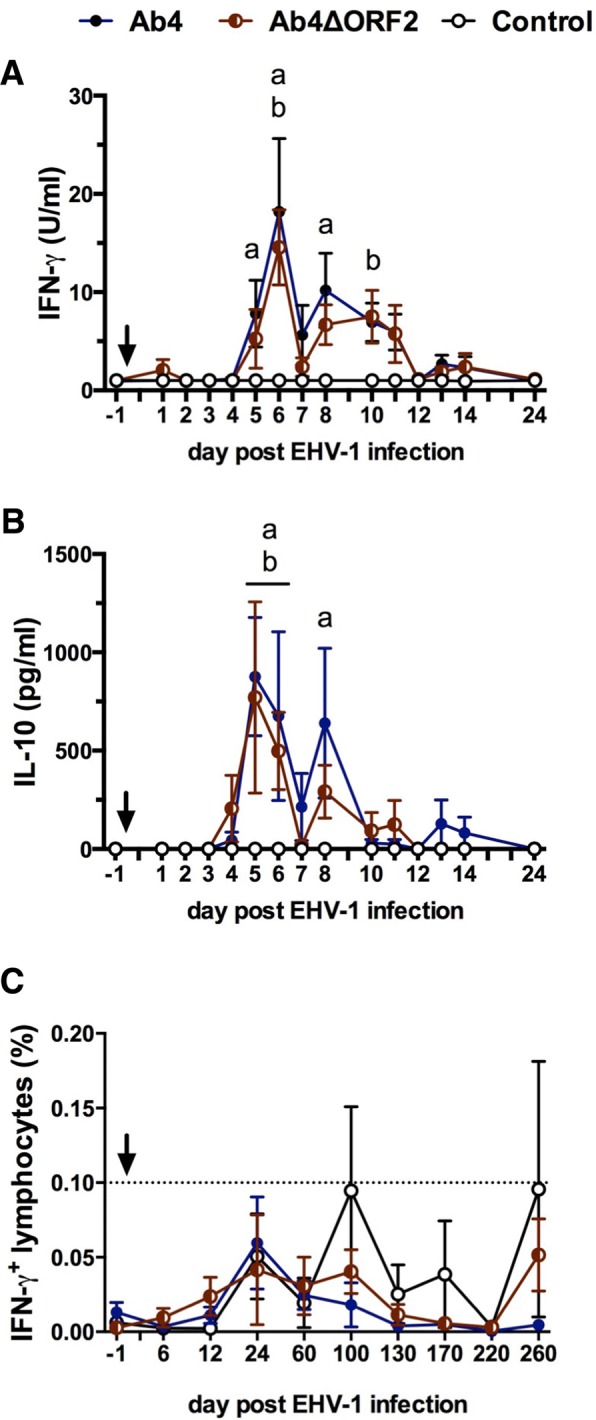
Cellular immune responses to infection in PBMC. Three groups of horses (*n* = 8/group) were either not infected (control), infected with the neuropathogenic EHV-1 strain Ab4 (Ab4) or infected with its ORF2 deletion mutant (Ab4ΔORF2) on day 0 (arrow). PBMC were isolated before (day − 1) and after infection (day 1–24) and re-stimulated ex vivo for 48 h with EHV-1 (strain Ab4). Cytokine production (**a** and **b**) was analyzed in supernatants by a fluorescent bead-based multiplex assay. All values are cell culture medium control corrected. Mean and standard errors of secreted IFN-γ (**a**) and IL-10 (**b**) are shown. EHV-1 stimulated PBMC were also fixed, stained for intracellular IFN-γ and analyzed by flow cytometry. Total EHV-1 specific IFN-γ producing lymphocytes are displayed (**c**). Values have been corrected by the respective medium control of each horse. The dotted horizontal line (**c**) represents a cutoff of 0.1% IFN-γ + lymphocytes. PBMC controls kept in medium alone typically result in values below this cutoff value. Significant differences between groups are marked: **a** = Ab4 vs. control, **b** = Ab4ΔORF2 vs. control, **c** = Ab4 vs. Ab4ΔORF2

Cellular immune responses of all groups were analyzed by ex vivo re-stimulation of PBMC with the EHV-1 strain Ab4. Between 5 and 10 days pi, IFN-γ secretion was elevated in horses of both the Ab4 group (days 5, 6, 8 pi, *p* < 0.05) and the Ab4ΔORF2group (days 6, 10 pi, *p* < 0.05) (Fig. 5a) compared to the control group. The IL-10 secretion displayed a similar biphasic response with the IL-10 peak on d5 pi slightly preceding the maximal IFN-γ response on d6pi. Both infected groups had greater IL-10 secretion from re-stimulated PBMC on days 5 and 6 pi (*p* < 0.05), and IL-10 was again elevated at d8 pi in the Ab4 group (*p* < 0.01) while the Ab4ΔORF2 group had a non-significant trend at d8 pi (Fig. 5b) compared to the control group. At later time points (d60-d260 pi) induction of cytokine secretion from PBMC in response to ex vivo re-stimulation was not observed for any group. Significant IL-4 and IL-17 secretion was not induced at any time (data not shown). Secretion of IFN-α was induced by the in vitro infection of PBMC at all time points, but there was no difference between the three groups of horses during the study (data not shown).

In addition, EHV-1 specific T-cells were analyzed by flow cytometry for intracellular IFN-γ, IL-4, IL-10 and IL-17 production. IFN-γ production by lymphocytes in PBMC were overall low and not different between the three groups for the duration of the study (Fig. 5c). IL-4, IL-10 or IL-17 were not induced in lymphocytes after ex vivo re-stimulation with EHV-1 (data not shown).

### Cytokine responses in serum

Cytokine measurements in serum were overall similar between the control and the infected groups with a few exceptions. IFN-α was increased in serum early after infection on days 2 and 4 pi in the Ab4ΔORF2 group compared to the control group (*p* < 0.05, Additional file 1: Figure S1A). In the Ab4 group there was a non-significant trend of increased serum IFN-α on d2 pi. It remained at a similar level to uninfected controls for the remainder of the measurements in both infected groups. Serum sCD14 was elevated in the Ab4 group compared to the control group at d3 pi (*p* < 0.01). In addition, sCD14 was higher in the control group compared to the Ab4ΔORF2 group at d13 pi (*p* < 0.05) but was increased in the Ab4ΔORF2 group compared to both, Ab4 and the controls at d24 pi, while it was still significantly higher in Ab4 than in controls at that day (*p* < 0.05, Additional file 1: Figure S1B). The levels of serum IFN-γ did not differ between the three groups at any point during the experiment (Additional file 1: Figure S1C). IL-4 and IL-17 concentrations were overall low in serum and without any differences between the three groups (data not shown).

## Discussion

An experimental infection with Ab4ΔORF2 deletion mutant virus in comparison to the parental neuropathogenic Ab4 virus was performed in horses to characterize the effects of the ORF2 gene product on EHV-1 infection and host immunity. Clinical signs, nasal shedding, and viremia induced by both viruses were evaluated to identify differences in virulence. Host immunity was analyzed locally and systemically summarizing the influence of both viruses on innate and adaptive cellular immunity and antibody induction. Overall, the infection of the horses with the parent Ab4 strain in the present study induced similar clinical signs, virus shedding, and viremia as previously described for EHV-1 infections of susceptible hosts [14, 42, 55]. Most importantly, the deletion of ORF2 reduced the virulence of the neuropathogenic EHV-1 Ab4 virus. This was evident by clearly reduced magnitude of the initial fever and significantly lower viral shedding in horses infected with the Ab4ΔORF2 deletion mutant virus. The reduced virulence observed here for Ab4ΔORF2 was comparable to the shorter duration of pyrexia, decreased magnitude and duration of virus shedding but unaffected viremia after infection of ponies with Ab4ΔORF1/2 compared to Ab4 in an earlier approach [42]. Differences in the magnitude of the fever induced by Ab4 infection may be based on individual differences between the ponies used by Soboll et al. [42] and the Icelandic horses used in the present study. Importantly, the reduction of virus shedding after deleting the ORF2 gene from the Ab4 virus versus using an Ab mutant deprived of ORF1 and ORF2 in the previous study [42] should be directly compared: Soboll et al. quantified EHV-1 DNA by PCR, while we used a plaque assay for virus isolation from nasal secretion, which measures infectious virus and thus correlates more directly with viral shedding and the ability of the infected horse to infect other horses.

The virulence factor ORF2 impacted the infection of the upper respiratory tract and local innate immune response to EHV-1 infection. In this study, we provided a comprehensive evaluation of intranasal cytokine and antibody secretion to longitudinally describe local innate and adaptive host immune responses during EHV-1 infection and potential differences induced by the ORF2 gene product. The onset of the local innate immune response against Ab4 was observed during virus shedding as determined by isolation of infectious virus from nasal discharge on days 2–4 pi. Intranasal innate immunity was characterized by sequential increases of the cytokines IFN-α (peak on d2pi), IFN-γ (peak on d3pi), and IL-10 (peak on d4pi) followed by a quick decline of all three cytokines. The nasal secretion of bioactive IFN was correlated with the time of EHV-1 shedding in a prior experimental infection study by Edington [56]. Here, we identified that high IFN-α in nasal secretion positively correlates with EHV-1 shedding in the Ab4 infected horses. In comparison, Ab4ΔORF2 infection induced low viral shedding combined with decreased IFN-α secretion. It has been demonstrated earlier, that equine IFN-α detected by the mAb pair used for quantification here, is also bioactive [57]. This suggests that intranasal IFN-α serves as an early danger signal during EHV-1 infection and, as previously pointed out by Randall [58], likely supports the induction of an antiviral state in the infected mucosal tissues. Notably, local IFN-γ and IL-10 induction was similar in both infected groups, Ab4ΔORF2 and Ab4, indicating that the ORF2 gene product does not influence these two immune regulatory cytokines.

Besides the short-term and pointed IFN-α, IFN-γ, and IL-10 secretion, elevated sCD14 concentrations were locally induced by EHV-1 between days 2–5 pi, while the horses were shedding virus. Peak intranasal sCD14 concentrations were slightly reduced in Ab4ΔORF2 infected horses supporting a possible direct or indirect influence of the ORF2 gene on intranasal sCD14 secretion. Lipopolysaccharide (LPS) binding sCD14 is produced by exocytosis and shedding of CD14 from monocyte or macrophage cell surfaces [59–61] and has a neutralizing, anti-inflammatory role for protection against LPS-induced cell death [62, 63]. The exact mechanisms of sCD14 induction after EHV-1 infection still needs to be unraveled in future approaches. However, EHV-1 was described to infect monocytes in vitro and in vivo [7, 64, 65] and the nasal sCD14 increase could thus result from a stress response of EHV-1 infected macrophages of the upper respiratory tract or reflect responses related to phagocytosis of virus and infected cells.

Overall, these findings point towards a role of ORF2 in the lytic virus replication cycle in the nasopharyngeal epithelia, which supported the release of infectious virions and increased the local viral load and nasal shedding. Nevertheless, the transmission of EHV-1 to mononuclear cells resulting in cell-associated viremia [7, 66] appears to be independent of ORF2. Here, viremia in the Ab4ΔORF2 and Ab4 groups was similar. Additionally, in vitro replication of Ab4ΔORF2 or Ab4ΔORF1/2 in RK13 cells and equine dermal cells (NBL-6) was not impaired compared to Ab4 [42]. Thus, the impact of ORF2 on nasal virus replication can only be shown after in vivo infection of the mucosal epithelial tissue including an intact local innate immune response.

In contrast to the clear reduction of virulence and intranasal IFN-α and sCD14 secretion, the immunogenicity of EHV-1 was not influenced by the deletion of the ORF2 gene from Ab4, with the only exception of a slightly earlier onset of IgG1 antibody responses in serum and during the second intranasal IgG1 peak in Ab4 infected horses. Local EHV-1 specific antibodies were detectable intranasally within 3–4 days after infection at low values. At this time, antibody responses were not yet detectable in serum. This very early nasal Ig secretion was similarly composed of different IgG isotypes and seems to be derived from local cells, such as *lamina propria* memory B-cells or those located in the regional lymph nodes. It should again be pointed out that all horses in this study were infected with EHV-1 about 1.5 to 2.5 years prior to the EHV-1 infection performed here. This confirmed the transient nature of protective immunity to EHV-1 described earlier [3, 27]. Although our horses did not mount a rapid and sufficiently high immune response to be protected from the EHV-1 infection performed here, they likely still had EHV-1 specific memory B-cells in their lymphatic tissues. Binding of viral antigens may have stimulated these memory B-cells to quickly secrete EHV-1 specific antibodies in low, but detectable quantities 3–4 days after infection.

Interestingly, this initial local IgG response waned quickly and was followed by peaks of higher local antibody responses that were dominated by IgG1 between d8–12 pi and IgG4/7 after d14 pi. Our findings clearly indicate that mucosal immune responses against the EHV-1strain Ab4 are dominated by IgG antibodies. Intranasal EHV-1 specific IgG antibodies are secreted in waves for several months post infection. The reason for the wave-like local antibody pattern is not yet clear. It requires further evaluation to identify if they could reflect local subclinical EHV-1 re-activation stages or other mechanisms. In contrast, the nasal EHV-1 specific IgA response after Ab4 infection was of overall low magnitude with slightly higher values for some horses that were infected with Ab4ΔORF2 during d8–14 pi. EHV-1 specific IgA was not detectable at later time points supporting that IgA is neither a major nor a long-lasting antibody during the mucosal EHV-1 specific immune response. This finding is contradictory to Breathnach [67], who found significant induction of nasal EHV-1 specific IgA together with IgG isotypes 2 weeks after primary infection with the neuropathogenic EHV-1 strain Army 183. Both, Breathnach [67] and our analysis here were based on the same monoclonal IgA antibody, BVS2, but used different assays for antibody detection. Future research could analyze if the observed difference in the EHV-1 specific IgA response are related to viral strain differences or if they have other reasons.

Robust systemic EHV-1 specific antibody responses were found in serum by d8 pi and were still detectable at high values by 9 months after infection with Ab4 or Ab4ΔORF2. Similar to nasal secretions, IgG1 was the dominating isotype of the early systemic response to EHV-1 infection, while IgG4/7 maintained the long-term response for more than 9 months. Similar serum antibody kinetics and IgG isotypes were previously reported after experimental EHV-1 infection with different EHV-1 strains including Ab4 and NY03 [42, 44]. This report exceeds the longitudinal observation period of previous articles of monitoring the immune response for weeks [67, 68] to 2–3 months [42, 69] after EHV-1 infection.

The glycoproteins gC and gD of EHV-1 are entry receptors of EHV-1 and highly immunogenic [70, 71]. In horses, gC and gD specific serum Ig and IgG4/7 highly correlate with EHV-1 serum neutralization titers [43]. Here, EHV-1 gC and gD specific antibodies were measured as representative markers for local and systemic EHV-1 specific immune responses with anti-gD responses closely resembling those of anti-gC. Equine IgG1 and IgG4/7 antibodies expressed effector functions such as strong complement activation and a high affinity to bind to IgG Fc-receptors in vitro [72]. EHV-1 specific IgG1 and IgG4/7 were thus assumed to have neutralizing activity and mediate protection against EHV-1 infection [22, 52]. Furthermore, IgG1 and IgG4/7 have been associated with a Th1 dominated B-cell response against EHV-1 [43].

In contrast to the robust antibody induction, an EHV-1 specific T-cell response could not be detected after infection with Ab4 or Ab4ΔORF2. Similarly, only weak T-cell responses were observed in former studies after experimental infection with EHV-1 Ab4 or NY03 [42, 44, 69]. However, we and others demonstrated increased EHV-1 specific cytotoxic T-cells frequencies or IFN-γ producing T-cells during viremia in older horses [13, 73], a few weeks after infection [74–76], or in horses that experienced an EHM outbreak and several vaccinations afterwards [52]. This suggests that EHV-1 specific T-cell activation is not consistently observed after infection, can be a slow process, and may require several immune recognition and activation events with EHV-1 or an EHV vaccine to develop substantial EHV-1 specific T-cell immunity. In the present study, a detectable cellular response was limited to EHV-1 re-stimulation ex vivo, occurred during viremia, and was represented by increased secretion of IL-10 and IFN-γ from PBMC. Likely, the transient stimulations of cytokine secretion from PBMC resulted from an innate response to the viral infection of circulating immune cells. It is believed that EHV-1 down-regulates cellular immunity and thereby allows the establishment of cell-associated viremia, which is a prerequisite for infection of endothelial cells prior to developing EHM or abortions [27, 66]. Current modified-live vaccines containing deletions of ORF2 and other genes [38, 40], similar to Ab4ΔORF2, fail to induce T-cell immunity and thus do not prevent viremia [22, 23, 52]. The EHV-1 genes responsible for the down-regulation of T-cell immunity still need to be identified to achieve robust cellular host immunity against EHV-1.

## Conclusions

The ORF2 gene of the EHV-1 strain Ab4 is a virulence factor which contributes to fever and viral shedding but does not affect viremia or adaptive host immunity against the virus (Graphical abstract). Infection with EHV-1 Ab4 and its ORF2 deletion mutant induces high local and systemic antibody responses dominated by early IgG1 and long-lasting IgG4/7 isotypes. Local EHV-1 specific IgA antibodies are only a minor component of the mucosal immune response. Antibody responses dominate EHV-1 immunity while T-cell immunity is overall low.

## Additional file

Additional file 1: **Figure S1.** Cytokines in serum after infection with EHV-1 strain Ab4 or its deletion mutant Ab4ΔORF2. Horses (*n* = 8/group) were infected with one of two EHV-1 strains (Ab4) or Ab4ΔORF2 at day 0 or kept as uninfected controls. Serum was sampled at several times before (days − 8, − 1) and after (day 1- day 24) infection, cytokines and the inflammatory marker sCD14 were evaluated with fluorescent bead-based assays. Mean and standard errors for IFN-α (A), sCD14 (B), and IFN-γ (C) in the serum are displayed. Significant differences between groups are marked: a = Ab4 vs. control, b = Ab4ΔORF2 vs. control, c = Ab4 vs. Ab4ΔORF2. (JPG 332 kb)

## Abbreviations

Ab4ΔORF2: Deletion mutant of Ab4 devoid of open reading frame 2
CI: Confidence interval
d pi: Days post infection
EHM: Herpesvirus myeloencephalopathy
EHV-1: Equine herpesvirus type 1
gB/C/D: Envelope glycoprotein B/C/D
IFN: Interferon
Ig: Immunoglobulin
IL: Interleukin
LPS: Lipopolysaccharide
MFI: Median fluorescent intensities
NS: Nasopharyngeal secretion
ORF: Open reading frame
PBMC: Peripheral blood mononuclear cells
PBS: Phosphate buffered saline
PFU: Plaque-forming units
PMA: Phorbol12-myristate13-acetate
RK13: Rabbit kidney 13 cells
sCD14: Soluble cluster of differentiation 14
