# Supplementary figures and images for: Deletion of the ORF2 gene of the neuropathogenic equine herpesvirus type 1 strain Ab4 reduces virulence while maintaining strong immunogenicity
Source: BMC Vet Res. 2018 Aug 22;14:245. doi: 10.1186/s12917-018-1563-4 (PMC6106926; doi:10.1186/s12917-018-1563-4)

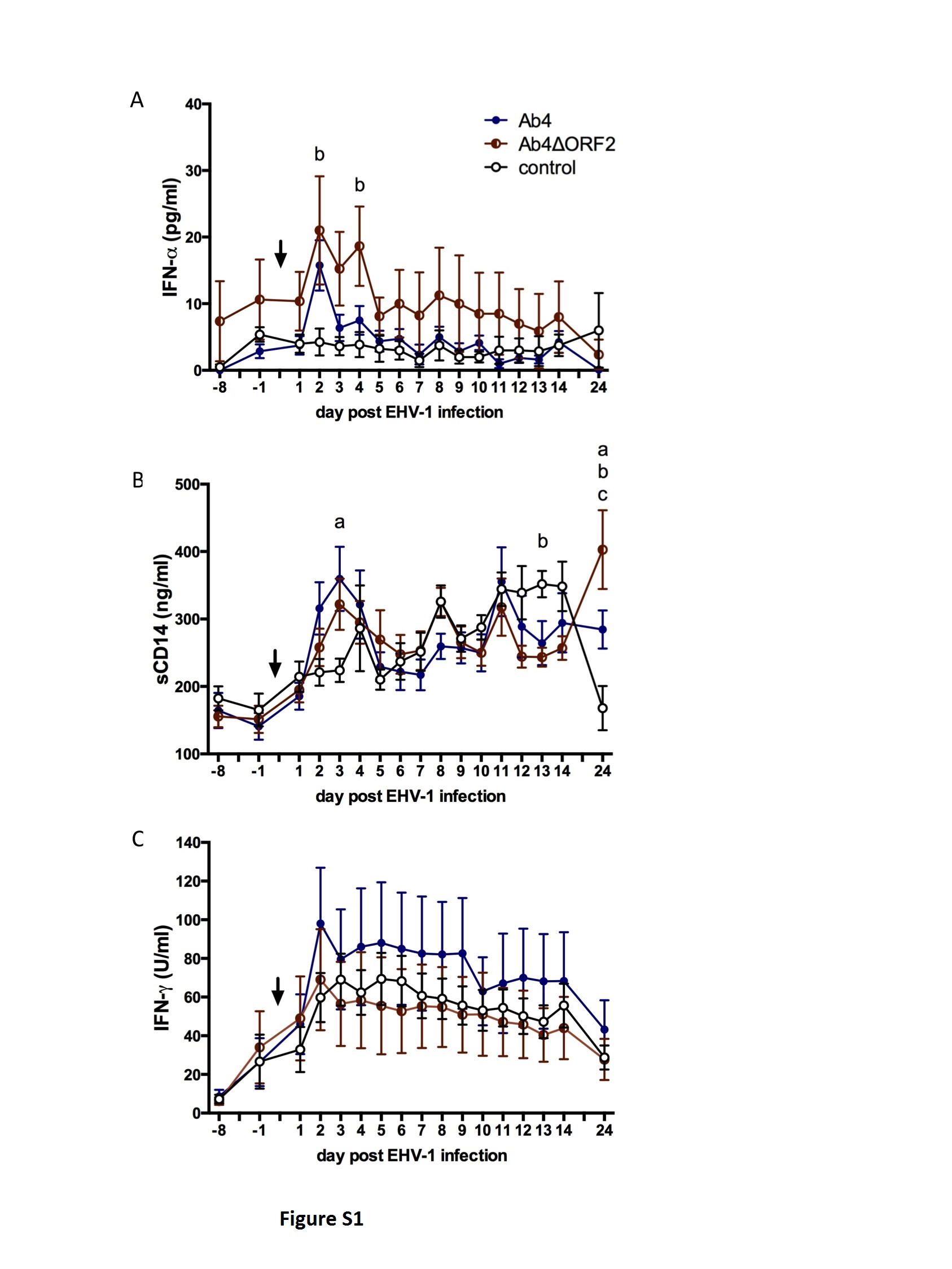

Supplement: Supplementary file 1 — Figure S1. Cytokines in serum after infection with EHV-1 strain Ab4 or its deletion mutant Ab4ΔORF2. Horses (n = 8/group) were infected with one of two EHV-1 strains (Ab4) or Ab4ΔORF2 at day 0 or kept as uninfected controls. Serum was sampled at several times before (days − 8, − 1) and after (day 1- day 24) infection, cytokines and the inflammatory marker sCD14 were evaluated with fluorescent bead-based assays. Mean and standard errors for IFN-α (A), sCD14 (B), and IFN-γ (C) in the serum are displayed. Significant differences between groups are marked: a = Ab4 vs. control, b = Ab4ΔORF2 vs. control, c = Ab4 vs. Ab4ΔORF2. (JPG 332 kb) [file 12917_2018_1563_MOESM1_ESM.jpg]
